# Associations between metabolic and structural retinal parameters and depression in individuals with type 2 diabetes

**DOI:** 10.1186/s40942-026-00800-x

**Published:** 2026-01-27

**Authors:** Frederik Nørregaard Pedersen, Lonny Stokholm, Noemi Lois, Dawei Yang, Carol Y. Cheung, Rafael Simó, Tunde Peto, Frans Pouwer, Jakob Grauslund

**Affiliations:** 1 Department of Ophthalmology, Odense University Hospital, J. B. Winsløws Vej 4 5000, Odense C, Denmark; 2 Department of Clinical Research, University of Southern Denmark, Odense, Denmark; 3 OPEN – Open Patient data Explorative Network, Odense University Hospital & University of Southern Denmark, Odense, Denmark; 4 Wellcome-Wolfson Institute for Experimental Research, School of Medicine, Dentistry and Biomedical Sciences, Queen’s University, Belfast, Northern Ireland UK; 5 Department of Ophthalmology and Visual Sciences, The Chinese University of Hong Kong, Hong Kong Special Administrative Region, Hong Kong , China; 6 Department of Endocrinology, Hospital Universitari Vall d’Hebron, Vall d’Hebron Research Institute (VHIR) and CIBERDEM (ISCIII), Barcelona, Spain; 7 Centre for Public Health, Queen’s University Belfast, Belfast, UK; 8 Department of Psychology, University of Southern Denmark, Odense, Denmark; 9 Steno Diabetes Center Odense, Odense University Hospital, Odense, Denmark

**Keywords:** Depression, Type 2 diabetes, Mood disorder, IDS-SR-30, Retinal parameters

## Abstract

**Background:**

We aim to examine whether a spectrum of retinal parameters, are associated with depression in individuals with type 2 diabetes.

**Method:**

We performed a cross sectional study of elderly patients with type 2 diabetes. Depression was measured with the Self-rated Inventory of Depressive Symptomatology 30 questionnaire, with scores ≥ 18 indicating depression. We evaluated retinal oxygen saturation, and retinal microvascular structures in individuals with type 2 diabetes and whether these were associated with increased risk of depression. Structural retinal parameters were measured in fundus photographs, and OCT-angiography scans, whereas retinal oxygen saturation was assessed with Oxymap Model T1. Main outcome was risk of depression. Multivariable logistic regression was used to investigate the association between retinal parameters and depression.

**Results:**

We included 134 individuals with type 2 diabetes, of whom 22% had depression. Median (with interquartile range) age and duration of diabetes were 72 (69-76) and 19 (13-23) years and 70.2% were male. Depression was associated with higher retinal arteriolar oxygen saturation in the lower temporal quadrant (OR 1.71 95% CI 1.07-2.75 per SD increase). We found no significant association with retinal vessel width, tortuosity, density, fractal dimension, area of foveal avascular zone or area of non-perfusion.

**Conclusion:**

We found some retinal metabolic but not vascular structures to be associated with depression, which therefore only partly support the notion that vascular factors may play a role in the pathogenesis of depression.

**Trial registration:**

The study was registered at http://www.clinicaltrials.gov before initiation (NCT04610749).

**Supplementary Information:**

The online version contains supplementary material available at 10.1186/s40942-026-00800-x.

## Introduction

Depression is a mood disorder characterized by several symptoms that alter functionality of an individual [1]. It is a common psychiatric disease with an estimated lifetime prevalence of 15% which causes emotional, cognitive and behavioral changes [2]. Depression can be regarded as a common psychiatric complication of type 2 diabetes that is linked to poor health outcomes and higher mortality rates [3]. A systematic review of longitudinal studies indicated that the association between depression and diabetic retinopathy (DR) is probably bidirectional [4]. The burden of being diagnosed with DR or having impaired vision or other associated diabetes-related complications may contribute to depression. On the contrary, depression may increase the risk for incident DR by sub-optimal medication use and subsequent higher HbA_1c_ levels, a less healthy diet, and lower physical activity levels [5, 6]. The heterogeneity within the disease of depression also needs to be taken into account, as the clinical presentation and treatment response various according to subtypes of depression including melancholic and atypical depression. According to the Diagnostic and Statistical Manual of Mental Disorders-5, atypical depression is characterized by a reactivity in mood and increased appetite and hyperinsomina whereas melancholic depression is described as non-reactivity in mood, insomnia, loss of appetite, and weightless [1]. Recent studies have shown that atypical depression is associated with metabolic syndrome and inflammation, whereas melancholic depression is primarily associated with hyperactivity of the hypothalamic-pituitary-adrenal axis, which subsequently may affect the vasculature. Although multiple factors may explain the relationship between type 2 diabetes and depression, a shared vascular pathogenesis has been proposed as a common denominator, with endothelial dysfunction being associated with depressive symptoms [7]. If the vascular hypothesis of depression is correct, conceivably, it could be hypothesized that non-invasive parameters of the retina could be associated with depressive symptoms. Studies have reported an association between retinal parameters and depression with reduced retinal arteriolar tortuosity [8], and wider retinal arterioles [9], but results have been inconsistent with no association found between retinal vessels width and symptoms of depression in other studies [10, 11]. Most studies were comparing people with and without diabetes and depression, except for one cross-sectional study. That study found an association between wider retinal arterioles in individuals with type 2 diabetes and depression, but did not evaluate other relevant retinal parameters e.g. vessel tortuosity, density or fractal dimension [9].

We aim to investigate the association between retinal metabolism, vascular structure and depression in individuals with type 2 diabetes as well as the relationships between retinal parameters and melancholic and atypical depression symptomatology.

## Subjects, material and methods

### Subjects

We conducted a cross-sectional study with participants recruited from the Funen Diabetes Database (FDDB); a regional database of individuals diagnosed with diabetes [12]. Potential participants were invited by a secure digital mailbox which is mandatory for individuals above 15 years of age to sign up for. If exempted from this service e.g. blindness or physical impairment, a formal letter was sent. Individuals who were willing to participate were screened for inclusion and exclusion criteria (excl. criteria iii-v) by telephone for efficiency purposes. We included individuals of 65 years or more with type 2 diabetes for five years or longer. We excluded individuals in case of (i) prior history of diagnosis of stroke or any neurodegenerative disease, (ii) known present retinal neurodegeneration due to previous laser photocoagulation, glaucoma or diabetic macular edema, (iii) presence of other eye disorders (beside DR and complications here of) affecting vision, (iv) Refractive error ≥6 diopters (D), (v) media opacities impeding retinal imaging and (vi) severe systemic illness or personal circumstances, which prevents completion of the study protocol.

### Systemic examination

All clinical visits were performed at the department of Ophthalmology, Odense University Hospital, Denmark between 25 November 2020 and 25 February 2022. General medical and ophthalmologic history were obtained. Duration of diabetes was obtained from FDDB, and in cases of discrepancy between the medical notes and the registry, the first registration date of type 2 diabetes diagnosis was used to calculate the duration of disease. Current use of antihypertensive, cholesterol lowering, glucose lowering, and antidepressant drugs were noted. Smoking was categorized as former, never and current user. Four-hour fasting blood samples were collected in order to examine HbA_1c_, lipid levels, and kidney function. Furthermore, a morning urine sample was collected to measure albumin excretion. With the individual sitting, brachial blood pressure was measured with Omron M6 (HEM-7001-E, Hoofddorp, The Netherlands), and mean arterial pressure was calculated as BP_d_+(BP_d_+BP_s_) / 3 where BP_d_ and BP_s_ are the diastolic and systolic blood pressure, respectively. Waist circumference, height and weight were measured and body mass index (BMI) calculated.

### Depressive symptoms score

Depressive symptoms were measured using the Self-rated Inventory of Depressive Symptomatology (IDS-SR-30) questionnaire, which consists of 30 items that rate the nine symptoms domain used to define major depressive episodes (DSM-IV criteria) as well as items to define atypical and melancholic symptoms. All items are rated on a scale from 0 (symptom is not present) to 3 (strongest impairment) and the total score ranging from 0 to 84 points (only 28 of the 30 items are rated). In the present study, the IDS-SR-30 was used primarily as a dichotomous variable with a cut-off point of 18 or above which indicates the presence of relevant depressive symptomatology [13]. The IDS-SR-30 can also be used to distinguish between melancholic and atypical forms of depression. We used a previous definition of the subtypes developed by the STAR*D research group based on selected items of the IDS-SR-30 [14, 15]. On a continuous scale of either atypical or melancholic depressive symptomatology, we investigated the association with alterations in retinal metabolic and vascular parameters.

### Ophthalmologic evaluation

#### Visual acuity, fundus examination and color fundus photography

Best-corrected visual acuity (BCVA) was obtained first, using Early Treatment Diabetic Retinopathy Study charts at 4 m (Precision Vision, Illinois, USA). Then, fundus examination was undertaken after pupillary dilation with tropicamide 10 mg/ml and phenylephrine 10%, followed by 45-degree 7-field color fundus photography (TRC-50DX fundus camera, Topcon, Tokyo, Japan). DR grading was carried out by a certified grader according to the International Clinical Diabetic Retinopathy Disease Severity Scale, with each eye categorized as level 0 (no DR), 1 through 3 (mild, moderate and severe none-proliferative DR, respectively), or 4 (proliferative DR) [16].

#### Retinal oximetry

**Fig. 1:**
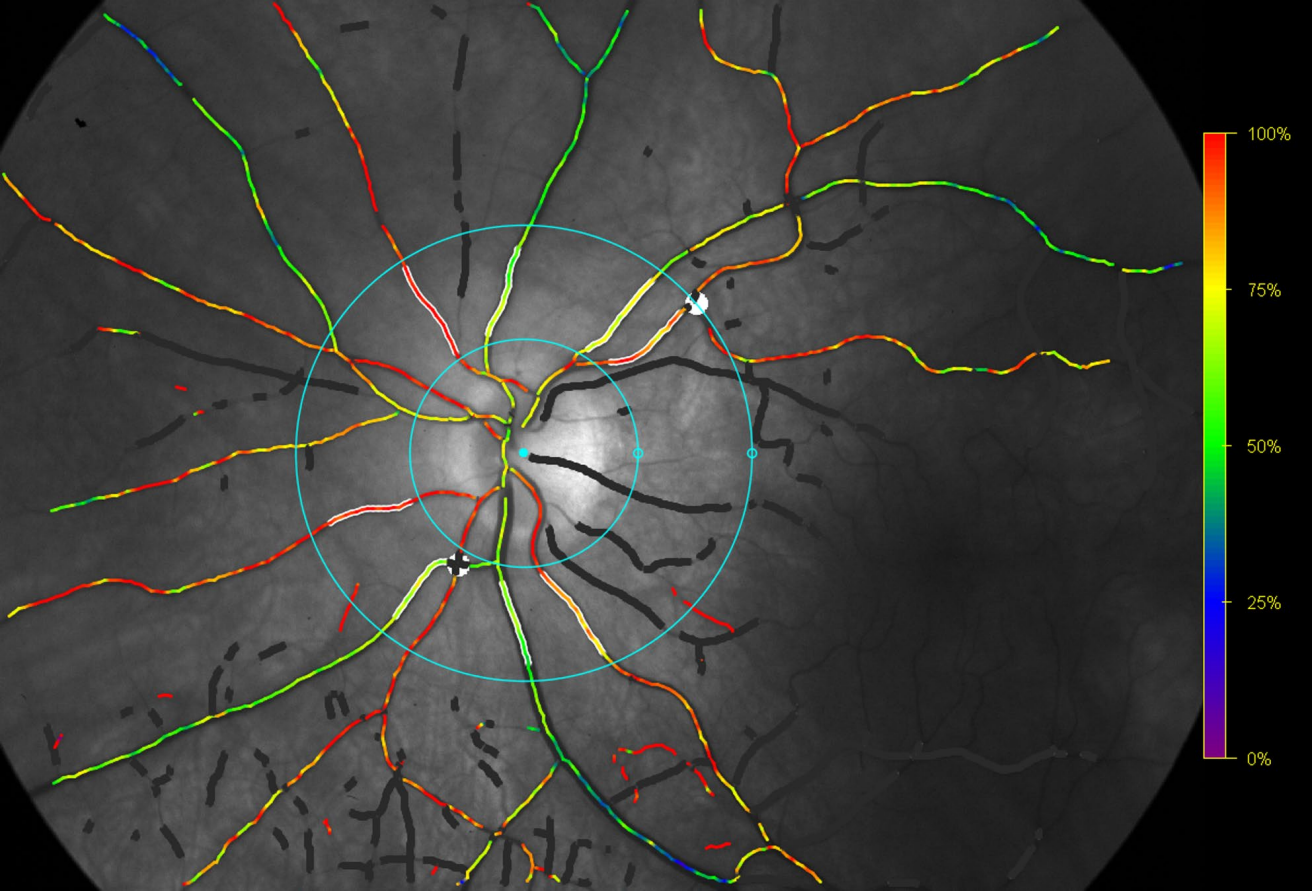
Retinal vessel oxygen measurements using the Oxymap Model T1. Overlaid colour map represents oxygen saturations in retinal vessels. The traced vessel representing the four retinal quadrants are highlighted within the circles and excluded vessel segments are covered in white

Retinal vessel oxygen saturation measurements were performed using the Oxymap Model T1 (Oxymap, Iceland), utilizing 50-degree disc centered images [17]. Two concentric circles (1.5 and 3 times diameter of optic disc) were positioned around the optic disc. The largest venule and arteriole in each quadrant were marked in length between 50 and 200 μm in order to measure the mean retinal oxygen saturation as well as perform quadrant analysis. Images with a quality score below five were excluded according to the Oxymap software (Fig. 1).

#### OCT-A parameters

Macula centered 4.5x.4.5 mm OCT-angiography (OCT-A) images were obtained with DRI OCT Triton (Topcon, Topcon Europe Medical B.V., The Netherlands). MATLAB (MathWorks, Natick, MA) was used for quantification of retinal microvasculature from OCT-A images [18]. The size of the foveal avascular zone (FAZ), vessel density, and fractal dimension in the superficial capillary plexus (SCP) and deep capillary plexus (DCP) were all retinal parameters of interest. Scans with image quality below 40, according to Topcon software, were excluded.

#### Retinal vessel analysis based on CPF

**Fig. 2:**
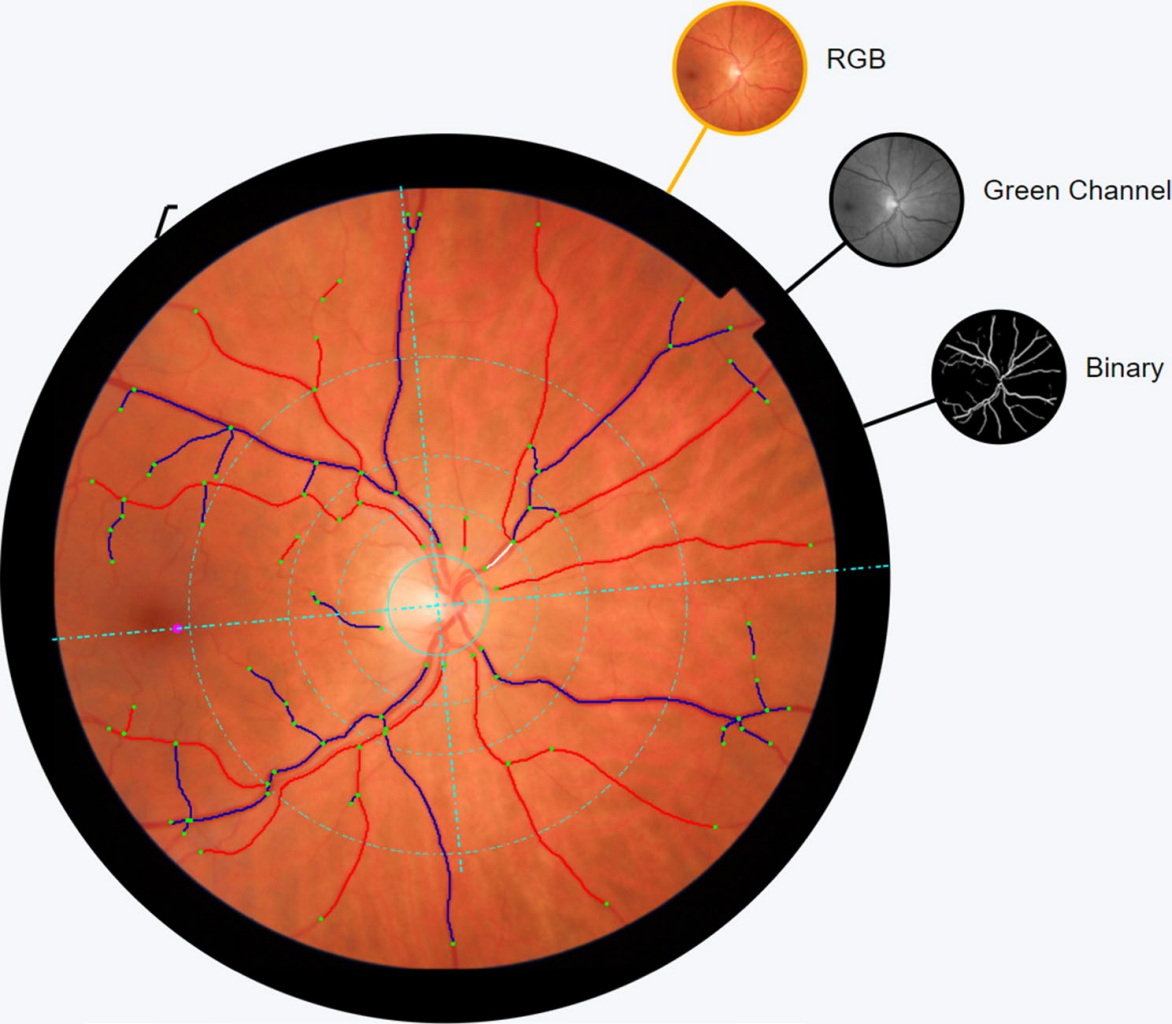
Example of the VAMPIRE-Web software used to measure retinal vessel width, density, tortuosity and fractal dimension. Venules are delineated with blue whereas arterioles are outlined with red colours. White lines indicate excluded vessels

VAMPIRE-Web (Vessel Assessment and Measurement Platform for Images of the Retina, Universities of Dundee and Edinburgh, UK) [19], a semiautomatic program was used to measure retinal vessel width, density, tortuosity and fractal dimension in 45-degree disc centered, color fundus photos (TRC-50DX fundus camera, Topcon, Tokyo, Japan). In cases of vessel mislabeling or misdetection, the annotation was manually corrected. The retinal vessel width was calculated as the central retinal artery and vein equivalent (CRAE and CRVE, respectively) through areas of one-half to one disc diameter from the disc margin [20]. Vessel density, tortuosity and fractal dimension were measured in the area of one-half disc diameter to two disc diameters from the disc margin (Fig. 2) [21].

### Statistical analysis

Descriptive statistics with categorical data are presented as numbers and percentages, and numerical data with medians and interquartile range (IQR). We applied Pearson chi-squared test (χ^2^) or Wilcoxonto test differences between groups (Table 1). We performed univariable logistic regression analysis to investigate the association between depression and baseline characteristics and retinal parameters (retinal oxygen saturation, vessel width, density, tortuosity, fractal dimension, area of FAZ and non-perfusion area) (Table 2). Cluster robust standard error was applied to the logistic regression model, when retinal parameters were investigated, while both eyes were included if eligible. We then performed multivariable logistic regression analysis adjusting for age, sex, HbA1c, diabetes duration, MAP, and history of depression (Table 2).

Lastly, we investigated whether presence of atypical or melancholic symptomatology on a continuous scale was associated with retinal vessel width, density, tortuosity, fractal dimension area of FAZ and non-perfusion area. This was performed in a multivariable mixed regression analysis with cluster robust standard error adjusted for age, sex, HbA1c, diabetes duration, MAP, and history of depression. Since the IDS-SR-30 scale was left-skewed and included zero, we tested whether the estimates differed between using the untransformed left-skewed scale and a square root-transformed scale. We found no substantial differences between the two models and thus present result from the analysis of the untransformed scale. We tested for interaction between age, sex and DR, which revealed interaction for DR and retinal oximetry, and therefore we performed stratified analysis (DR yes / DR no) (Supplementary material). Power calculation was performed in another study from our study unit [22]. P-values below 0.05 or 95% confidence intervals (CI) that did not include zero in the mixed regression model or CI that did not include 1.0 in the logistic regression model were considered statistically significant. Abovementioned statistics were performed with STATA version 17.0 (StataCorp LLC, College Station, TX, USA).

## Results

**Fig. 3:**
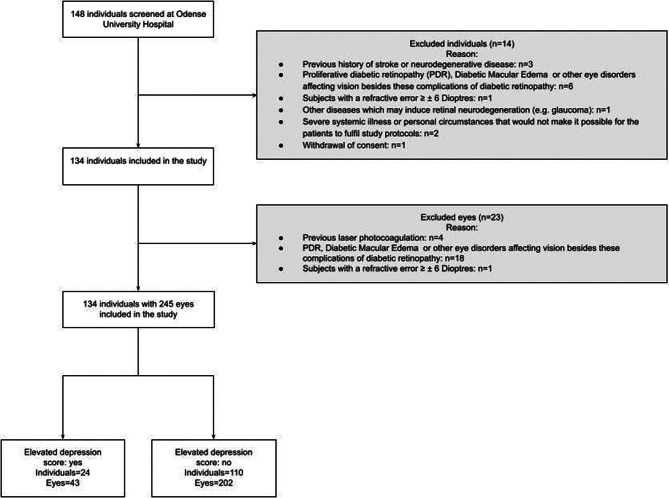
Flowchart of inclusion

We included 134 individuals with type 2 diabetes (Fig. 3), with a median age of 72 years (IQR: 69–76) and a median duration of diabetes of 19 years (IQR: 13–23). Of these participants, 70.2% were male. Based on IDS-SR-30 scores, 22% had depression (Table 1). Those with depression were more likely to have a previous history of depression (29% vs. 6%, *p* < 0.001), be current smokers (25% vs. 5% *p* = 0.004), have higher levels of total cholesterol (4.1 vs. 3.6 mmol/L, *p* = 0.021) and LDL cholesterol (2.0 vs. 1.5 mmol/L, *p* = 0.042), have an albumin excretion rate above 300 mg/g (17% vs. 1.8% *p* = 0.005) and be current users of antidepressants drugs (13% vs. 3%, *p* = 0.037). There was no difference in age, sex, civil status, BMI, waist circumference, BCVA or DR severity level between groups. Missing descriptive data included waist circumference (*n* = 1), total cholesterol (*n* = 1), LDL cholesterol (*n* = 2), estimated glomerular filtration rate (*n* = 1), and albumin excretion rate (*n* = 1). Of the 245 eyes examined, one person did not have retinal oxygen saturation measurement performed due to equipment failure. In addition, the VAMPIRE software were not able to calculate venular and arterial fractal in 4 and 33 eyes, respectively. Analysis of vessel width, density, fractal dimension area of the FAZ and area of non-perfusion was based on all included eyes.

**Table 1:** Characteristics of individuals and eyes with and without depression in people with type 2 diabetes

	Depression		*p*-value
	Yes	No	
Individuals, n	24	110	
Sex, male %(n)	66.7% (16)	70.9% (78)	0.68
Age, years median (IQR)	71.0 (69.0, 76.5)	72.0 (69.0, 75.0)	0.84
**Current partner status %(n)**			
Single	37.5% (9)	20.9% (23)	0.084
Married or living together	62.5% (15)	79.1% (87)	
History of ischemic heart disease yes %(n)	25.0% (6)	20.0% (22)	0.59
History depression yes %(n)	29.2% (7)	6.4% (7)	< 0.001
Diabetes duration (years), median (IQR)	19.5 (16.0, 23.0)	18.0 (13.0, 24.0)	0.43
Present DR yes %(n)	70.8% (17)	68.2% (75)	0.80
BMI (kg/m^2^), median (IQR)	29.3 (25.9, 34.2)	29.3 (26.2, 32.4)	0.95
**Smoking %(n)**			
Current	25.0% (6)	4.5% (5)	0.004
Never	33.3% (8)	40.0% (44)	
Former	41.7% (10)	55.5% (61)	
MAP (mmHg), median (IQR)	103.7 (94.5, 110.5)	101.3 (93.3, 108.0)	0.47
Waist circumference (Cm), median (IQR)	108.0 (98.0, 117.0)	106.0 (99.0, 116.0)	0.64
**Medication use %(n)**:			
Current use of antidepressants	12.5% (3)	2.8% (3)	0.037
Antihypertensive treatment	4.2% (1)	15.5% (17)	0.14
Statin treatment	75.0% (18)	82.7% (91)	0.38
Antidiabetic treatment			
Glucose lowering treatment excluding insulin	87.5% (21)	87.3% (96)	0.98
Insulin treatment	62.5% (15)	48.2% (53)	0.20
**Laboratory tests**:			
HbA_1c_ (mmol/L), median (IQR)	59.5 (50.5, 68.0)	55.0 (50.0, 62.0)	0.30
Total cholesterol (mmol/L), median (IQR)	4.1 (3.4, 5.0)	3.6 (3.1, 4.1)	0.021
LDL cholesterol (mmol/L), median (IQR)	2.0 (1.3, 2.7)	1.5 (1.2, 1.9)	0.042
HDL cholesterol (mmol/L), median (IQR)	1.3 (1.0, 1.6)	1.2 (1.1, 1.6)	0.89
Triglycerides (mmol/L), median (IQR)	1.8 (1.2, 3.1)	1.5 (1.0, 2.0)	0.11
eGFR (ml/min/1.73 m2), median (IQR)	76.0 (46.0, 90.0)	77.0 (58.0, 88.0)	0.91
**Albumin excretion rate (mg/g)**,** %(n)**			
<30	70.8% (17)	78.2% (86)	0.005
30–300	12.5% (3)	20.0% (22)	
>300	16.7% (4)	1.8% (2)	
Eyes included	43	202	
Pseudophakia, yes %(n)	30.2% (13)	31.7% (64)	0.85
BCVA ETDRS letters, median (IQR)	84.0 (80.0, 85.0)	84.5 (81.0, 86.0)	0.13
**Diabetic retinopathy**,** ICDR %(n)**			
Level 0	25.6% (11)	32.7% (66)	0.71
Level 1	23.3% (10)	19.8% (40)	
Level 2	46.5% (20)	45.0% (91)	
Level 3	4.7% (2)	2.5% (5)	

IQR: Interquartile range. ICDR: the International Clinical Diabetic Retinopathy. MAP: Mean arterial pressure. GDS-15: Geriatric depression scale. BMI: Body mass index. eGFR: estimated glomerular filtration. BCVA: Best corrected visual acuity. ETDRS: Early Treatment Diabetic Retinopathy Study

In a univariable logistic regression model, a previous history of depression was associated with depression (OR 6.06 95% CI 1.89–19.45, yes vs. no), total cholesterol (OR 1.59 95% CI 1.06–2.39 per 1 mmol/L increase), higher retinal arteriolar oxygen saturation in the lower temporal quadrant (OR 1.58 95% CI 1.08–2.30 per SD increase) and higher retinal venular oxygen saturation in the upper nasal quadrant (OR 1.48 95% CI 1.03–2.11 per SD increase) (Table 2). History of depression (OR 6.16 95% CI 1.8–20.8 pr. SD increase) and retinal arteriolar oxygen saturation in the lower temporal quadrant (OR 1.71 95% CI 1.07–2.75 pr. SD increase) was still statistical significant in the multivariable adjusted model. There was no association between retinal vessel width, tortuosity, vessel density, fractal dimension, area of FAZ, area of non-perfusion, mean retinal vessel oxygen saturation and depression.

**Table 2:** Uni- and multivariable logistic regression model with OR and 95% CI for baseline characteristics and retinal parameters associated with depression

	Increment	Univariable logistic regression OR (95% CI)	*p*-value	Multivariable logistic regression OR (95% CI)	*p*-value
**Individuals characteristics**					
Sex	Male vs. female	1.22 (0.48–3.13)	0.68	1.13 (0.42–3.09)	0.80
Age	1 year	1.00 (0.91–1.09)	0.98	1.02 (0.93–1.13)	0.60
Civil status	Partner vs. no partner	2.27 (0.88–5.84)	0.09	2.02 (0.73–5.54)	0.17
History of Ischemic heart disease	Yes vs. no	1.33 (0.47–3.75)	0.59	1.45 (0.46–4.57)	0.53
History of depression	Yes vs. no	6.06 (1.89–19.45)	0.002	6.16 (1.8–20.8)	0.003
History of smoking	Yes vs. no	1.33 (0.53–3.38)	0.55	1.32 (0.47–3.70)	0.59
Diabetes duration	5-year	1.06 (0.80–1.42)	0.67		
Present DR	Yes vs. no	1.13 (0.43–2.98)	0.80	0.99 (0.33-3.00)	0.99
MAP	5 mmHg	1.07 (0.88–1.30)	0.52	1.06 (0.85–1.31)	0.62
BMI	1%-point	1.00 (0.92–1.09)	0.99	1.00 (0.91–1.09)	0.98
Abdominal circumference	Pr. 1 cm	1.00 (0.97–1.03)	0.87	1.00 (0.97–1.04)	0.92
**Laboratory tests**					
Total cholesterol	1 mmol/L	1.59 (1.06–2.39)	0.03	1.63 (1.00-2.65)	0.05
HDL-cholesterol	1 mmol/L	0.89 (0.31–2.56)	0.84	0.78 (0.20–2.97)	0.71
LDL-cholesterol	1 mmol/L	1.61 (0.99–2.62)	0.06	1.65 (0.92–2.97)	0.09
Triglycerides	1 mmol/L	1.49 (1.00-2.23)	0.05	1.43 (0.89–2.29)	0.14
eGFR	1 ml/min/1.73 m2	1.00 (0.98–1.02)	0.84	0.99 (0.97–1.02)	0.48
HbA1c	10 mmol/L	1.18 (0.82–1.69)	0.38	1.10 (0.75–1.63)	0.62
Albumin excretion rate	Above vs. below 30 ml/min/1.73 m^2^	1.48 (0.55–3.97)	0.44	1.43 (0.49–4.21)	0.51
**Use of medication**					
Antidepressants	Yes vs. no	5.05 (0.95–26.74)	0.057		
Insulin use	Yes vs. no	0.56 (0.23–1.38)	0.21	0.58 (0.18–1.88)	0.36
Glucose lowering excl. insulin	Yes vs. no	1.05 (0.35–3.13)	0.83	0.95 (0.29–3.11)	0.93
Lipid lowering use	Yes vs. no	0.63 (0.22–1.79)	0.38	0.70 (0.22–2.27)	0.56
Antihypertensive	Yes vs. no	0.24 (0.03–1.88)	0.17	0.20 (0.02–1.71)	0.14
**Retinal parameters**	Increment	Univariable logistic regression^*^ OR (95% CI)	p-value	Multivariable logistic regression^*^ OR (95% CI)	p-value
Retinal vessel oxygen saturation					
Mean arterial saturation	SD	1.01 (0.96–1.06)	0.69	1.02 (0.96–1.08)	0.64
Mean venular saturation	SD	1.03 (0.99–1.07)	0.16	1.03 (0.98–1.08	0.30
Quadrant arterial analysis					
Upper nasal	SD	1.15 (0.80–1.67)	0.45	1.18 (0.80–1.73)	0.41
Lower nasal	SD	0.80 (0.48–1.34)	0.39	0.94 (0.65–1.37)	0.76
Upper temporal	SD	0.92 (0.68–1.25)	0.60	0.86 (0.63–1.19)	0.38
Lower temporal	SD	1.58 (1.08–2.30)	0.02	1.71 (1.07–2.75)	0.02
Quadrant Venular analysis					
Upper nasal	SD	1.48 (1.03–2.11)	0.03	1.38 (0.92–2.07)	0.12
Lower nasal	SD	1.15 (0.79–1.67)	0.47	1.04 (0.69–1.57)	0.19
Upper temporal	SD	1.06 (0.74–1.51)	0.74	1.04 (0.68–1.57)	0.86
Lower temporal	SD	1.30 (0.88–1.92)	0.19	1.38 (0.90–2.10)	0.14
OCT-A parameters macular region					
Superficial capillary plexus					
Foveal avascular zone	SD	0.89 (0.67–1.19)	0.42	0.88 (0.66–1.17)	0.38
Vascular density					
Circle	SD	1.02 (0.73–1.43)	0.92	1.05 (0.74–1.49)	0.79
Superior sector	SD	1.03 (0.71–1.48)	0.88	1.06 (0.72–1.56)	0.77
Right sector	SD	1.14 (0.86–1.52)	0.35	1.16 (0.87–1.55)	0.31
Inferior sector	SD	0.98 (0.74–1.31)	0.91	1.00 (0.76–1.33)	0.98
Left sector	SD	0.94 (0.66–1.33)	0.73	0.97 (0.67–1.39)	0.85
Non-perfusion area					
Circle	SD	0.93 (0.67–1.30)	0.68	0.91 (0.64–1.28)	0.58
Superior sector	SD	0.91 (0.64–1.28)	0.58	0.88 (0.61–1.26)	0.48
Right sector	SD	0.81 (0.60–1.10)	0.18	0.81 (0.59–1.10)	0.17
Inferior sector	SD	1.04 (0.79–1.37)	0.77	1.03 (0.78–1.37)	0.81
Left sector	SD	1.07 (0.76–1.50)	0.71	1.04 (0.73–1.49)	0.83
Fractal Dimension	SD	0.95 (0.69–1.30)	0.73	0.97 (0.70–1.35)	0.88
Deep capillary plexus					
Foveal avascular zone	SD	0.91 (0.66–1.26)	0.57	0.90 (0.66–1.24)	0.53
Vascular Density					
Circle	SD	0.93 (0.69–1.24)	0.60	0.94 (0.71–1.24)	0.65
Superior sector	SD	1.02 (0.77–1.37)	0.88	1.05 (0.80–1.40)	0.71
Right sector	SD	1.20 (0.95–1.53)	0.13	1.21 (0.95–1.54)	0.12
Inferior sector	SD	0.81 (0.59–1.13)	0.22	0.81 (0.59–1.12)	0.21
Left sector	SD	0.89 (0.65–1.22)	0.46	0.90 (0.66–1.22)	0.49
Non-perfusion area					
Circle	SD	1.06 (0.77–1.46)	0.71	1.08 (0.77–1.50)	0.66
Superior sector	SD	0.94 (0.68–1.29)	0.70	0.92 (0.68–1.25)	0.61
Right sector	SD	0.79 (0.59–1.06)	0.12	0.80 (0.59–1.08)	0.15
Inferior sector	SD	1.35 (0.96–1.88)	0.09	1.38 (0.97–1.95)	0.07
Left sector	SD	1.13 (0.82–1.54)	0.46	1.13 (0.83–1.52)	0.44
Fractal dimension	SD	0.97 (0.68–1.39)	0.88	1.02 (0.72–1.43)	0.92
Retinal structural parameters (Vampire analysis)					
CRAE	SD	0.96 (0.68–1.36)	0.81	0.93 (0.64–1.34)	0.68
CRVE	SD	1.27 (0.87–1.84)	0.22	1.17 (0.80–1.71)	0.43
Arteriolar tortuosity	SD	0.95 (0.63–1.44)	0.81	1.0 (0.64–1.56)	0.99
Venular tortuosity	SD	1.07 (0.79–1.46)	0.65	1.12 (0.83–1.50)	0.46
Arteriolar density	SD	0.91 (0.61–1.35)	0.63	0.91 (0.63–1.31)	0.60
Venular density	SD	1.06 (0.69–1.63)	0.79	0.98 (0.67–1.43)	0.90
Arteriolar fractal dimension	SD	0.96 (0.70–1.31)	0.78	0.95 (0.67–1.35)	0.78
Venular fractal dimension	SD	1.14 (0.76–1.72)	0.52	1.06 (0.73–1.55)	0.76
Total fractal dimension	SD	1.00 (0.69–1.45)	1.00	0.98 (0.68–1.40)	0.90

^*^In analysis of retinal parameters cluster robust standard error was employed to the model while both eyes, if eligible, were included. The multivariate model included age, sex, HbA1c, diabetes duration, MAP, and history of depression. BMI: Body mass index. CRAE: Central retinal arterial equivalent. CRVE: Central retinal arterial equivalent. DR: Diabetic retinopathy. eGFR: estimated glomerular filtration. MAP: Mean arterial pressure. OR: Odds ratio. SD: standard deviation

Lastly, on a continuous scale of atypical or melancholic depressive symptomatology, we investigated the association with retinal metabolic and vascular parameters. We found an association between higher retinal arteriolar oxygen saturation in the lower temporal quadrant and melancholic depressive scores (β: 0.56 95% CI 0.09–1.30). We found a lower retinal venular saturation in the lower nasal quadrant and atypical depressive scores (β -1.39 95% CI -2.43-(-0.35)), but only in a subgroup of those with DR. Finally, we did not find any correlations between retinal vessel width, tortuosity, vessel density, fractal dimension, area of FAZ, area of non-perfusion with atypical or melancholic depression scores (Supplementary Tables 1–3).

## Discussion

We found almost one-fourth to have depression in patients with type 2 diabetes, which is in line with results of earlier studies [23]. Alterations in measures of retinal arteriolar and venular oxygen saturation levels were found to be associated with some extent to depression and higher melancholic and atypical depressive score.

A few individuals had a previous history of depression or were current users of antidepressant medication, but still a high proportion had relevant clinical depression scores based on IDS-SR-30. The prevalence of depression in patients with type 2 diabetes has varied significantly with estimates between 5.0 and 71.8% [24]. Multiple factors may lead to this differences between reported prevalence in which differences in study population, ethnicity, socioeconomic status, accessibility to health care and method to assess depression may be the causes. In the present study we used the IDS-SR-30 which has previous been validated to be suitable for clinical use, and comparable with other well-known instruments for screening for depression including the Hamilton Rating Scale for Depression [25]. Still the accuracy of the tests can vary between populations and should be taken into account interpretation the results with risk of overestimating or underestimating the prevalence. Furthermore, there can be an overlap between symptoms of diabetes distress and depression e.g. fatigue which could result in false positive scores [26]. Nevertheless, the high-observed prevalence of depression in this study, indicate that a high proportion of individuals with type 2 diabetes express undetected depressive symptomatology, which may lead to poorer health outcomes.

It is over two decades since Alexopoulos et al. described the vascular depression hypothesis proposing that cerebrovascular disease may predispose geriatric depressive syndromes [27]. Since then, some evidence has been presented that vascular disease may be related to depression, but data has overall been inconsistent [28]. Endothelial dysfunction, characterized by circulating inflammatory biomarkers, has been associated with elevated depression scores, which again strengthens the hypothesis that microvascular abnormalities play a role in the pathogenesis of depression [7].

We found higher retinal arteriolar saturation in the lower temporal quadrant to be associated with depression, which we speculate to be associated with retinal flow. The retinal vessel oxygen saturation measurements are inversed affected by the retinal blood flow, meaning a decreased blood flow equals a higher saturation measurement [29]. Depression in elderly is associated with increased hypertension, diabetes and atherosclerosis, which subsequently leads to vascular wall hypertrophy, reduced arterial lumen, and reduced blood flow, subsequently resulting in a higher retinal arterial saturation [30]. Even though this may be a plausible pathophysiological pathway, we were not able to find a difference in retinal vessel width between the two groups.

We did not find a clear association between retinal oxygen saturation and melancholic or atypical depressive symptomatology. The heterogeneity within depression and variety in clinical presentation may also come to present in the retina meaning that certain depressive symptomatology present with different alterations in the retina, which may also be the reason for our findings of altered retinal saturation. It is important to address that previous studies have shown an adverse effect on retinal oxygen saturation as well as several retinal structural parameters in individuals with diabetes. These adverse effects may also be present to some extent in our study population. But, as we only investigated individuals with type 2 diabetes and the groups were comparable between diabetes duration, presence of DR, Hb1Ac levels and blood pressure, these adverse retinal effects may be balanced between groups.

It is also important to mention that several of our results were not in line with the vascular depression hypothesis. We found no associations between depression and retinal vessel structure, which is opposite to previous findings. A cross-sectional study reported that wider retinal arterioles were associated with major depression in persons with type 2 diabetes and major depression (*n* = 43), compared to individuals with type 2 diabetes without depression (*n* = 49) and a healthy control group without diabetes or depression (*n* = 54) [9]. Even though that study reports a difference in arteriolar width, the statically method described does not compare individuals with type 2 diabetes and depression but all three groups simultaneously. Therefore, it does not directly assess the difference in a population of type 2 diabetes alone.

Strengths of this study include the standardized software programs to assess retinal vessel parameters and vessel saturation. Limitations include the lack of masking of depressive scores to the researcher performing retinal analysis, with the exception of OCT-A analysis. Furthermore we did not have data on socio-economic factors which may have a role in depression. To our knowledge, no prior study has investigated the association between oxygen saturation in the retinal vasculature in people with type 2 diabetes and depression. Even though these are novel findings, caution should be made when interpreting the data presented, with the potential of spurious associations due to multiple testing.

In conclusion, we report that oxygen saturation in the retinal vasculature is altered in individuals with depression and type 2 diabetes. Therefore, this study partly supports the vascular hypothesis, but further studies are warranted to investigate, how retinal parameters may be used as a proxy to identify people with depression. 

## Supplementary Information

Below is the link to the electronic supplementary material.

Supplementary Material 1

## Data Availability

Data is available upon reasonable request to the author.
