# Supplementary material for: Associations between metabolic and structural retinal parameters and depression in individuals with type 2 diabetes
Source: Int J Retina Vitreous. 2026 Jan 27;12:29. doi: 10.1186/s40942-026-00800-x (PMC12874965; doi:10.1186/s40942-026-00800-x)
Supplement: Supplementary file 1 — Supplementary Material 1 [file 40942_2026_800_MOESM1_ESM.docx]

Supplementary Data

S. Table1. Elevated atypical depression and melancholic scores associations with retinal vessel oxygen saturation.

S. Table2. Elevated atypical and melancholic depression scores associations with retinal structural parameters (OCT-A).

S. Table 3. Elevated atypical and melancholic depression scores associations with retinal structural parameters (Vampire analysis).

S. Table 4. Depression and retinal vessel oxygen saturation stratified by presence of DR

S.Table 1: Elevated atypical depression and melancholic scores associations with retinal oxygen saturation.

|  | Mean retinal saturation  %, β (95%CI) | | Arterial saturation quadrant analysis  %, β (95%CI) | | | | Venular saturation quadrant analysis  %, β (95%CI) | | | |
| --- | --- | --- | --- | --- | --- | --- | --- | --- | --- | --- |
|  | Arteriolar | Venular | Upper nasal | Lower Nasal | Upper Temporal | Lower Temporal | Upper nasal | Lower Nasal | Upper Temporal | Lower Temporal |
| Atypical depressive score | 0.02  (-0.54-0.59) | -0.01 (-0.98-0.96 | 0.14 (-0.58-0.86) | -0.42 (-1.57-0.73) | -0.36 (-0.99-0.26) | 0.70 (-0.02-1.41) | 0.13 (-0.90-1.18) | -0.45 (-1.50-0.60) | 0.04 (-1.16-1.23) | 0.14 (-1.12-1.40) |
| DR yes | -0.27 (-0.91-0.37) | -0.75 (-1.65-0.16) | -0.03 (-0.85-0.80) | -0.86 (-2.65-0.93) | -0.50 (-1.21-0.22) | 0.31 (-0.42-1.05) | -0.29 (-1.39-0.80) | **-1.39 (-2.43-(-0.35))*** | -0.71 (-1.89-0.46) | -0.41 (-1.59-0.78) |
| DR no | 0.35 (-0.53-1.23) | 1.00 (-0.76-2.76) | 0.22 (-0.78-1.21) | 0.09 (-1.02-1.20) | -0.10 (-1.22-1.03) | 1.13 (-0.17-2.43) | 0.43 (-1.39-2.26) | 1.53 (-0.53-3.58) | 1.45 (-0.60-3.50) | 0.80 (-1.81-3.41) |
| Melancholic  Depressive score | -0.01 (-0.43-0.40) | -0.23 (-0.92-0.46) | 0.19 (-0.33-0.72) | -0.29 (-1.06-0.48) | -0.46 (-0.96-0.03) | **0.56 (0.03-1.08)*** | -0.15 (-0.75-0.44) | -0.34 (-1.21-0.52) | -0.42 (-1.27-0.43) | -0.38 (-1.28-0.52) |
| DR yes | 0.17 (-0.39-0.73) | -0.17 (-0.98-0.65) | 0.42 (-0.33-1.17) | -0.34 (-1.60-0.92) | -0.12 (-0.78-0.54) | **0.70 (0.09-1.30)*** | 0.03 (-0.86-0.93) | -0.26 (-1.27-0.74) | -0.35 (-1.43-0.73) | -0.19 (-1.21-0.83) |
| DR no | -0.07 (-0.63-0.50) | **-**0.05 (-1.10-1.00) | 0.18 (-0.40-0.75) | -0.12 (-0.83-0.58) | **-0.91 (-1.64-(-0.17))*** | 0.56 (-0.49-1.60) | 0.23 (-0.77-1.22) | -0.57 (-2.13-1.00) | 0.20 (-0.93-1.31) | -0.35 (-2.33-1.63) |

Cluster robust standard error linear mixed model analysis adjusted for age, sex, HbA1c, diabetes duration, MAP, and history of depression. β: coefficient of the linear mixed model regression. P-values below 0.05 indicated with *. CI: Confidence interval. DR: Diabetic retinopathy. MAP: Mean arterial pressure.

S.Table 2: Elevated atypical and melancholic depression scores associations with retinal structural parameters.

|  | **Superficial capillary plexus** | |  | **Deep capillary plexus** | |
| --- | --- | --- | --- | --- | --- |
|  | **Atypical depressive symptoms** | **Melancholic depressive symptoms** |  | **Atypical depressive symptoms** | **Melancholic depressive symptoms** |
| FAZ | 0.007 (-0.03-0.04) | 0.004 (-0.02-0.02) |  | -0.008 (-0.03-0.01) | -0.005 (-0.02-0.01) |
| Vascular Density %, β (95%CI) |  |  |  |  |  |
| Circle | 0.004 (-0.005-0.013) | 0.002 (-0.005-0.008) |  | -0.001 (-0.006-0.003) | 0.0006 (-0.003-0.004) |
| Superior sector | 0.003 (-0.008-0.013) | -0.00004(-0.008-0.008) |  | -0.001 (-0.007-0.005) | 0.001 (-0.003-0.005) |
| Right sector | 0.006 (-0.006-0.020) | 0.007 (-0.002-0.025) |  | 0.002 (-0.005-0.009) | 0.003 (-0.001-0.008) |
| Inferior sector | 0.002 (-0.009-0.012) | 0.001 (-0.006-0.009) |  | -0.005 (-0.01-0.003) | -0.002 (-0.007-0.003) |
| Left sector | 0.004 (-0.007-0.01) | -0.002 (-0.01-0.008) |  | -0.001 (-0.008-0.006) | -0.0007 (-0.006-0.005) |
| Non-perfusion area 1mm^2^, β (95%CI) |  |  |  |  |  |
| Circle | -0.05 (-0.16-0.07) | -0.03 (-0.11-0.054) |  | 0.03 (-0.03-0.10) | -0.02 (-0.07-0.04) |
| Superior sector | -0.007 (-0.04-0.03) | -0.002 (-0.03-0.02) |  | 0.005 (-0.02-0.03) | -0.006 (-0.02-0.01) |
| Right sector | -0.02 (-0.06-0.02) | -0.03 (-0.06-(-0.001))* |  | -0.006 (-0.03-0.02) | -0.02 (-0.04-0.008) |
| Inferior sector | -0.006 (-0.04-0.025) | -0.004 (-0.03-0.02) |  | 0.03 (-0.003-0.06) | 0.005 (-0.02-0.03) |
| Left sector | -0.01 (-0.05-0.02) | 0.007 (-0.03-0.04) |  | 0.005 (-0.02-0.03) | 0.002 (-0.02-0.02) |
| Fractal dimension | 0.0002 (-0.001-0.001) | -0.0002 (-0.001-0.0009) |  | -0.00008 (-0.001-0.0009) | -0.0001 (-0.0009-0.0007) |

Cluster robust standard error linear mixed model analysis adjusted age, sex, HbA1c, diabetes duration, MAP, and history of depression. β: coefficient of the linear mixed model regression. P-values below 0.05 indicated with *. CI: Confidence interval. FAZ: Foveal avscualar zone.

S.Table 3: Elevated atypical and melancholic depression scores associations with retinal structural parameters (Vampire analysis).

|  | Vessel caliber  pixels, β (95%CI) | | Vessel Tortuosity | | Vessel Density  pixels, β (95%CI) | | Fractal Dimension | |
| --- | --- | --- | --- | --- | --- | --- | --- | --- |
|  | Arterioles | Venules | Arterioles | Venules | Arterioles | Venules | Arterioles | Venules |
| Atypical  Depression | -0.08 (-0.25-0.08) | 0.18 (-0.08-0.43) | 0.02 (-0.22-0.19) | -0.003 (-0.11-0.11) | -8.8 (-79.6-62.0) | 67.5 (-26.3-161.3) | -0.034 (-0.10-0.036) | 0.0006 (-0.02-0.02) |
| Melancholic  Depression | -0.08 (-0.22-0.06) | 0.10 (-0.12-0.32) | 0.04 (-0.16-0.24) | 0.07 (-0.03-0.18) | 1.4 (-61.9-64.7) | 10.2 (-70.4-90.8) | -0.02 (-0.08-0.04) | -0.01 (-0.04-0.01) |

Cluster robust standard error linear mixed model analysis adjusted age, sex, HbA1c, diabetes duration, MAP, and history of depression. β: coefficient of the linear mixed model regression. CI: Confidence interval. P-values below 0.05 indicated with *.

| **Retinal parameters** | Increment | Univariable logistic regression OR (95% CI) | p-value | Multivariable logistic regression OR (95% CI) | p-value |
| --- | --- | --- | --- | --- | --- |
| **DR yes** |  |  |  |  |  |
| Retinal vessel oxygen saturation |  |  |  |  |  |
| Mean arterial saturation | SD | 1.00 (0.93-1.06) | 0.92 | 1.01 (0.93-1.09) | 0.82 |
| Mean venular saturation | SD | 1.01 (0.97-1.06) | 0.58 | 1.02 (0.96-1.08) | 0.53 |
| Quadrant arterial analysis |  |  |  |  |  |
| Upper nasal | SD | 1.01 (0.94-1.09) | 0.76 | 1.17 (0.70-1.93) | 0.55 |
| Lower nasal | SD | 0.98 (0.92-1.03) | 0.36 | 0.89 (0.60-1.33) | 0.58 |
| Upper temporal | SD | 0.99 (0.94-1.04) | 0.69 | 0.90 (0.60-1.37) | 0.63 |
| Lower temporal | SD | 1.04 (0.98-1.09) | 0.21 | 1.40 (0.83-2.37) | 0.21 |
| Quadrant Venular analysis |  |  |  |  |  |
| Upper nasal | SD | 1.03 (0.99-1.07) | 0.10 | 1.39 (0.86-2.26) | 0.18 |
| Lower nasal | SD | 1.00 (0.97-1.04) | 0.88 | 0.93 (0.57-1.52) | 0.77 |
| Upper temporal | SD | 0.99 (0.96-1.03) | 0.64 | 0.97 (0.60-1.58) | 0.91 |
| Lower temporal | SD | 1.01 (0.97-1.04) | 0.69 | 1.30 (0.75-2.22) | 0.35 |
| **DR no** |  |  |  |  |  |
| Retinal vessel oxygen saturation |  |  |  |  |  |
| Mean arterial saturation | SD | 1.03 (0.95-1.11) | 0.47 | 1.04 (0.98-1.11) | 0.21 |
| Mean venular saturation | SD | 1.07 (0.98-1.16) | 0.13 | 1.06 (0.98-1.16) | 0.15 |
| Quadrant arterial analysis |  |  |  |  |  |
| Upper nasal | SD | 1.03 (0.98-1.08) | 0.30 | 1.37 (0.87-2.15) | 0.17 |
| Lower nasal | SD | 0.98 (0.88-1.08) | 0.69 | 1.06 (0.65-1.74) | 0.81 |
| Upper temporal | SD | 0.97 (0.91-1.04) | 0.44 | 0.71 (0.39-1.27) | 0.24 |
| Lower temporal | SD | 1.11 (1.00-1.24) | 0.055 | 2.38 (0.98-5.82) | 0.06 |
| Quadrant Venular analysis |  |  |  |  |  |
| Upper nasal | SD | 1.04 (0.98-1.09) | 0.19 | 2.03 (0.84-4.88) | 0.11 |
| Lower nasal | SD | 1.03 (0.97-1.10) | 0.28 | 1.58 (0.56-4.45) | 0.38 |
| Upper temporal | SD | 1.03 (0.98-1.08) | 0.23 | 1.50 (0.81-2.77) | 0.20 |
| Lower temporal | SD | 1.04 (0.99-1.10) | 0.15 | 1.32 (0.66-2.64) | 0.43 |

S.Table 4. Depression and retinal vessel oxygen saturation stratified by presence of DR

Multivariable logistic regression analysis with cluster robust standard error adjusted for age, sex, HbA1c, diabetes duration, MAP, and history of depression. CI: Confidence interval. DR: Diabetic retinopathy. MAP: Mean arterial pressure. OR: odds ratio.
